# Genetic feature diversity of *KRAS*-mutated colorectal cancer and the negative association of DNA mismatch repair deficiency relevant mutational signatures with prognosis

**DOI:** 10.1016/j.gendis.2024.101245

**Published:** 2024-02-26

**Authors:** Ruichuan Shi, Yu Cheng, Jin Wang, Na Song, Ying Chen, Zan Teng, Ling Xu, Yunpeng Liu, Xiaotian Zhao, Qiuxiang Ou, Peng Yang, Rui Ma, Yiting Sun, Jinglei Qu, Xiujuan Qu

**Affiliations:** a Department of Medical Oncology, The First Hospital of China Medical University, Shenyang, Liaoning 110001, China; b Geneseeq Research Institute, Nanjing Geneseeq Technology Inc., Nanjing, Jiangsu 210032, China

*KRAS* mutations occur in approximately 40% of metastatic colorectal cancer (CRC), leading to disrupted hydrolysis of guanosine triphosphate and tumor cell proliferation.^1^ Genetic features and clinical outcomes of CRCs depend on *KRAS* mutation subtypes,^2^, ^3^, ^4^ and molecular biomarkers for prognosis prediction are under development. We supposed that mutational signatures offering an additional layer of genomic information might aid in understanding the differences in treatment efficacy among *KRAS*-mutated CRCs.

In this study, next-generation sequencing was performed on tumor tissue/liquid biopsies using a panel covering 425 cancer-related genes (Table S1; Supplementary Material and Methods). A total of 116 CRCs with various *KRAS* aberrations and 73 CRCs without *KRAS* aberrations were retrospectively enrolled at the First Hospital of China Medical University between March 2017 and August 2022 (Fig. S1A). The clinical characteristics of *KRAS*-mutated CRCs are summarized in Table S2. The diversity of genetic features was investigated in CRCs with and without *KRAS* mutations, and we further developed a mutational signature-based prognosis indicator for progression-free survival (PFS) and overall survival (OS) in first-line therapy.

Of 116 *KRAS*-mutated CRC samples, common *KRAS* aberrations included *KRAS*^*G12D*^ (32%), *KRAS*^*G12V*^ (21%), and *KRAS*^*G13D*^ mutations (15%), whereas *KRAS*^*G12C*^ mutations accounted for only 7% (Fig. 1A). Their genomic profiles were shown in Fig. S1B. The prevalence of mutated RAS pathway, defined as having any altered genes in the RAS pathway except for *KRAS*, was less common in *KRAS*^*G13X*^ CRCs than in *KRAS*^*G12X*^ CRCs (0.0% *vs.* 21.2%, *P* = 0.04; Fig. 1B) and in CRCs harboring *KRAS* alterations other than *KRAS*^*G12X*^ and *KRAS*^*G13X*^ (the Other subgroups) (0.0% *vs.* 25.0%, *P* = 0.03; Fig. 1B). Mutated RTK pathway was also more prevalent in the Other subgroup when compared with CRCs harboring *KRAS*^*G12X*^ (72.2% *vs.* 41.2%, *P* = 0.02; Fig. S1C). The genomic profiles of 73 *KRAS* wild-type CRCs are shown in Fig. S2A. Mutated RAS pathway was more prevalent in *KRAS* wild-type than in *KRAS*-mutated CRCs (38.4% *vs.* 19.8%, *P* < 0.01; Fig. S2B), due to the enriched *BRAF* mutations in *KRAS* wild-type CRCs (16.4% *vs.* 4.3%, *P* < 0.01; Fig. S2C). Of 97 *KRAS*-mutated CRC samples eligible for mutational signature identification, 62 (63.9%) were detected with at least one type of DNA mismatch repair deficiency (dMMR)-related mutational signature (Fig. S3).

**Figure 1:**
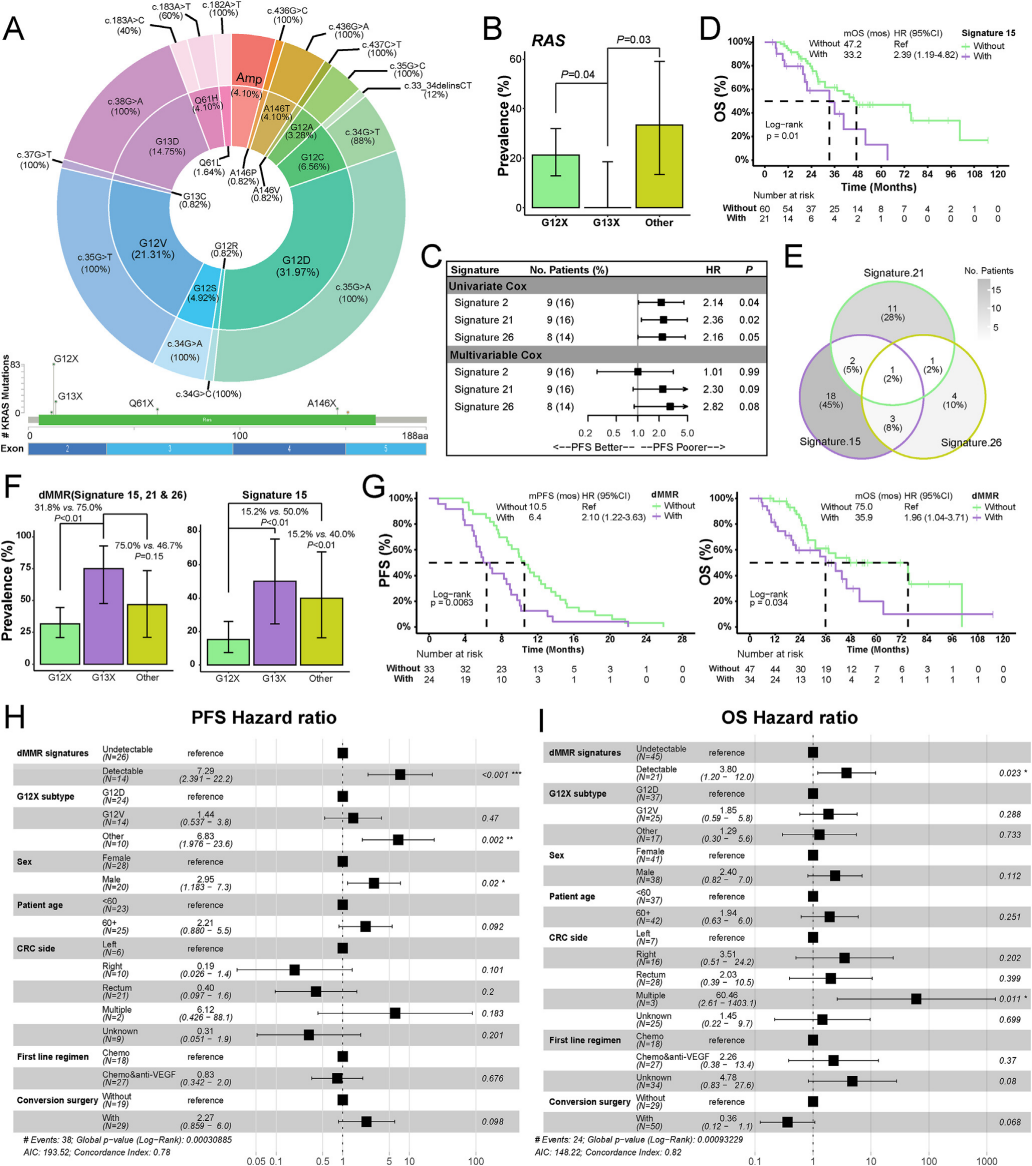
*KRAS* aberrations in colorectal cancer and the dMMR signature combination related to prognosis. **(A)** The proportion of each *KRAS* aberration subtype. **(B)** The prevalence of mutated RAS signaling pathway in patients with *KRAS*^*G12X*^, *KRAS*^*G13X*^, and *KRAS* aberrations other than *KRAS*^*G12X*^ and *KRAS*^*G13X*^ (the Other subgroup). **(C)** The dMMR-related mutational signatures 21 and 26 were associated with potentially poorer progression-free survival in first-line therapy. **(D)** The dMMR-related mutational signature 15 was associated with inferior overall survival. **(E)** Forty *KRAS*-mutated patients were identified with the dMMR signature combination of signature 15, 21, and 26, and patients with multiple dMMR-related mutational signatures were rarely observed. **(F)** The dMMR signature combination and mutational signature 15 were enriched in *KRAS*^*G13X*^ patients, in comparison with patients with *KRAS*^*G13X*^ and *KRAS* aberrations other than *KRAS*^*G12X*^ and *KRAS*^*G13X*^. **(G)***KRAS*-mutated patients with detectable dMMR signature combination had inferior progression-free survival and overall survival than those without. **(H, I)** The dMMR signature combination was strongly associated with inferior progression-free survival and overall survival when adjusting for *KRAS*^*G12X*^ subtype, sex, patient age, colorectal cancer side, first-line regimen, and conversion surgery. dMMR, DNA mismatch repair deficiency.

Fifty-seven of 116 *KRAS*-mutated CRCs had available PFS data for first-line therapy, including 23 and 32 patients who received chemotherapy and chemotherapy combined with anti-VEGF agents, respectively (the remaining 2 patients: unknown). None of the *KRAS* mutation subtypes, concomitant mutations, or mutated signaling pathways were associated with PFS in first-line treatment. Mutational signatures 21 and 26 were associated with inferior PFS in both univariate analyses (signature 21, hazard ratio/HR: 2.36, 95% confidence interval/CI: 1.12–4.98; signature 26, HR: 2.16, 95% CI: 1.01–4.64; Fig. 1C) and multivariable analyses adjusting for sex, patient age, *KRAS* mutation subtype, CRC side, first-line regimen, and conversion surgery (signature 21, HR: 2.30, 95% CI: 0.87–6.13; signature 26, HR: 2.82, 95% CI: 0.87–9.18; Fig. 1C). Interestingly, both signature 21 and signature 26 were related to DNA mismatch repair deficiency (dMMR). No significant differences in OS were observed across various *KRAS* mutation subtypes (Fig. S4A), whereas only signature 15, a dMMR-related mutational signature, was associated with worse OS (HR: 2.39, 95% CI: 1.19–4.82; Fig. 1D). However, signature 6, which was another dMMR-related signature, was not significantly associated with PFS (HR: 0.99, 95% CI: 0.56–1.76) or OS (HR: 1.10, 95% CI: 0.57–2.13).

Of 40 patients with detectable prognosis-related dMMR signatures, patients with more than one dMMR-related signature were rarely observed (*n* = 7, 17%; Fig. 1E). The dMMR signature combination including signatures 15, 21, and 26 was more prevalent in patients with *KRAS*^*G13X*^, when compared with patients harboring *KRAS*^*G12X*^ (75.0% *vs.* 31.8%, *P* < 0.01; Fig. 1F) and other *KRAS* alterations (75.0% *vs.* 46.7%, *P* = 0.15; Fig. 1F), due to the higher prevalence of signature 15 in *KRAS*^*G13X*^ patients than in *KRAS*^*G12X*^ patients (50.0% *vs.* 15.2%, *P* < 0.01; Fig. 1F). Additionally, the signature combination was more common in *KRAS*-mutated CRCs with mutated TGFβ pathway than in those without (56.8% *vs.* 31.7%, *P* = 0.02; Fig. S4B). Compared with 33 *KRAS*-mutated CRCs without the dMMR signature combination, 24 *KRAS*-mutated patients with the dMMR signature combination had inferior PFS (HR: 2.10, 95% CI: 1.22–3.63) and poorer OS (HR: 1.96, 95% CI: 1.04–3.71) in first-line treatment (Fig. 1G). In the multivariable Cox regression models controlling for sex, patient age, *KRAS* mutation subtype, CRC side, first-line chemotherapy regimen, and receiving conversion surgery or not, dMMR signature combination remained significantly associated with worse PFS (HR: 2.51, 95% CI: 1.20–5.22; Fig. S4C) and worse OS (HR: 2.73, 95% CI: 1.19–6.27; Fig. S4D).

As *KRAS*^*G12X*^ patients accounted for approximately 70% of *KRAS*-mutated CRCs, prognostic molecular features were further investigated in three *KRAS*^*G12X*^ subgroups, including the *KRAS*^*G12D*^ subgroup, the *KRAS*^*G12V*^ subgroup, and the other *KRAS*^*G12X*^ subgroup. Our data demonstrated that *KRAS*^*G12V*^ patients had poorer OS than *KRAS*^*G12D*^ patients (HR: 2.51, 95% CI: 1.08–5.85; Fig. S5A). Under first-line chemotherapy, the other *KRAS*^*G12X*^ subgroup might have worse PFS than *KRAS*^*G12D*^ (HR: 4.17, 95% CI: 1.02–17.13; Fig. S5B), whereas significant differences were not observed in *KRAS*^*G12X*^ patients receiving chemotherapy combined with anti-VEGF agents (Fig. S5C). Prognosis-related signature 15 appeared to be more common in *KRAS*^*G12V*^ than in *KRAS*^*G12D*^ patients while it is not statistically significant (28.6% *vs.* 9.4%, *P* = 0.13; Fig. S5D). Compared with *KRAS*^*G12X*^ patients without the dMMR signature combination, those with detectable dMMR signature combination had significantly inferior PFS (adjusted HR: 7.29, 95% CI: 2.39–22.24; Fig. 1H) and OS (adjusted HR: 3.80, 95% CI: 1.20–12.01; Fig. 1I) in first-line therapy, when adjusting for *KRAS*^*G12X*^ subgroup, sex, age, CRC side, first-line chemotherapy regimen, and conversion surgery treatment. Nevertheless, among *KRAS*-mutated patients with non-G12X *KRAS* mutations, neither first-line therapy PFS (HR: 0.97, 95% CI: 0.36–2.61; Fig. S6) nor OS (HR: 0.89, 95% CI: 0.31–2.53; Fig. S6) exhibited significant differences between patients with and without the dMMR signature combination.

Our data demonstrated the diversity of molecular features among CRCs harboring *KRAS* aberrations. A dMMR signature combination of signature 15, 21, and 26 could identify advanced CRCs with worse first-line PFS and OS, especially in CRCs with *KRAS*^*G12X*^ mutations. This study has some limitations. First, this study is a single-center retrospective study with missing data about CRC position, first-line and subsequent treatment regimens, and PFS and/or OS. Thus, our results should be interpreted with caution, and prospective studies with larger sample sizes are required to confirm our findings. In addition, although a large sequencing panel covering 425 genes was applied in our study, we expected to use whole-exome sequencing to define mutational signature^5^ and validate the association between dMMR signature and prognosis. Moreover, due to the limited sample size of CRCs with non-G12X *KRAS* mutations, further studies were warranted, particularly for *KRAS*^*G13X*^ patients who had a relatively high prevalence of the dMMR signature combination.

## Ethics declaration

This study was approved by the Ethics Committee of the First Hospital of China Medical University (approval No. [2017]-236), and written informed consent was provided by each participant.

## Author contributions

XQ and JQ designed the study. RS, YC, JW, NS, YC, ZT, LX, YL, RM, and YS were responsible for patient recruitment and sample and data collection. XZ, QO, PY, RS, and XQ analyzed data and interpreted results. All authors wrote and reviewed the manuscript and approved the submitted version.

## Conflict of interests

Xiaotian Zhao, Qiuxiang Ou, and Peng Yang are employees of Nanjing Geneseeq Technology Inc., China. The remaining authors have nothing to disclose.

## Funding

This study was supported by grants from the 10.13039/501100018537National Science and Technology Major Project of the Ministry of Science and Technology of China (No. 2017ZX09304025), the Technological Special Project of Liaoning Province, China (No. 2019020176-JH1/103), and the High-level innovation and entrepreneurship team of Liaoning province's "Xing Liao Talents Program" (No. XLYC2008006).

## Data availability

The datasets used and/or analyzed in the current study are available from the corresponding author upon reasonable request.
