# Supplementary material for: Genetic feature diversity of KRAS-mutated colorectal cancer and the negative association of DNA mismatch repair deficiency relevant mutational signatures with prognosis
Source: Genes Dis. 2024 Feb 26;12(1):101245. doi: 10.1016/j.gendis.2024.101245 (PMC12053584; doi:10.1016/j.gendis.2024.101245)
Supplement: Multimedia component 1 [file mmc1.docx]

**Supplementary Tables**

| **Table S1. A total of 425 genes covered by the target next-generation sequencing panel** | | | | |
| --- | --- | --- | --- | --- |
| SEPT9 | CUX1 | GRIN2A | NBN | RICTOR |
| ABCB1 (MDR1) | CXCR4 | GRM3 | NCOR1 | RNF43 |
| ABCC2 (MRP2) | CYLD | GRM8 | NF1 | ROS1 |
| ADH1B | CYP19A1 | GSTM1 | NF2 | RPTOR |
| AIP | CYP2A13 | GSTM4 | NFE2L2 | RRM1 |
| AKT1 | CYP2A6 | GSTP1 | NFKBIA | RUNX1 |
| AKT2 | CYP2A7 | GSTT1 | NKX2-1 | RUNX1T1 |
| AKT3 | CYP2B6*6 | HDAC2 | NOTCH1 | SBDS |
| ALDH2 | CYP2C19*2 | HDAC9 | NOTCH2 | SDC4 |
| ALK | CYP2C9*3 | HGF | NOTCH3 | SDHA |
| AMER1 | CYP2D6 | HLA-A | NPM1 | SDHB |
| APC | CYP3A4*4 | HNF1A | NQO1 | SDHC |
| AR | CYP3A5 | HNF1B | NRAS | SDHD |
| ARAF | CYSLTR2 | HRAS | NRG1 | SETBP1 |
| ARID1A | DAXX | IDH1 | NSD1 | SETD2 |
| ARID1B | DDR2 | IDH2 | NTRK1 | SF3B1 |
| ARID2 | DENND1A | IFNA6 | NTRK2 | SGK1 |
| ARID5B | DHFR | IFNB1 | NTRK3 | SKP2 |
| ASCL4 | DICER1 | IFNE | NUTM1 | SLC34A2 |
| ASXL1 | DLL3 | IFNG | PAK3 | SLC3A2 |
| ATF1 | DNMT3A | IFNGR1 | PALB2 | SMAD2 |
| ATIC | DOT1L | IFNGR2 | PALLD | SMAD3 |
| ATM | DPYD | IGF1R | PARK2 | SMAD4 |
| ATR | DTL (CDT2) | IGF2 | PARP1 | SMAD7 |
| ATRX | DUSP2 | IKBKE | PARP2 | SMARCA4 |
| AURKA | EGFR | IKZF1 | PAX5 | SMARCB1 |
| AURKB | EIF1AX | IL7R | PBRM1 | SMO |
| AXIN2 | EP300 | INPP4B | PDCD1 (PD1) | SOCS1 |
| AXL | EPAS1 | IRF2 | PDCD1LG2 (PD-L2) | SOS1 |
| B2M | EPCAM | JAK1 | PDE11A | SOX2 |
| BAD | EPHA2 | JAK2 | PDGFRA | SPOP |
| BAI3 | EPHA3 | JAK3 | PDGFRB | SPRED1 |
| BAK1 | EPHA5 | JARID2 | PDK1 | SPRY4 |
| BAP1 | ERBB2 (HER2) | JUN | PGR | SRC |
| BARD1 | ERBB2IP | KDM5A | PHOX2B | SRSF2 |
| BAX | ERBB3 | KDR (VEGFR2) | PIK3C3 | SRY |
| BCL2 | ERBB4 | KEAP1 | PIK3CA | STAG2 |
| BCL2L11 (BIM) | ERCC1 | KIF1B | PIK3CD | STAT3 |
| BCR | ERCC2 | KIT | PIK3R1 | STK11 |
| BIRC3 | ERCC3 | KITLG | PIK3R2 | STMN1 |
| BLM | ERCC4 | KLLN | PKHD1 | SUFU |
| BMPR1A | ERCC5 | KMT2A (MLL) | PLAG1 | TACC3 |
| BRAF | ESR1 | KMT2B | PLCB4 | TAP1 |
| BRCA1 | ETV1 | KMT2C | PLK1 | TAP2 |
| BRCA2 | ETV4 | KMT2D (MLL2) | PMS1 | TEK |
| BRD4 | ETV5 | KRAS | PMS2 | TEKT4 |
| BRIP1 | ETV6 | LHCGR | POLD1 | TERC |
| BTG2 | EWSR1 | LMO1 | POLD3 | TERT |
| BTK | EXT1 | LRP1B | POLE | TET2 |
| BUB1B | EXT2 | LYN | POLH | TGFBR2 |
| c11orf30 | EZH2 | LZTR1 | POT1 | THADA |
| CASP8 | EZR | MAP2K1 (MEK1) | PPARD | TMEM127 |
| CBL | FANCA | MAP2K2 (MEK2) | PPP2R1A | TMPRSS2 |
| CBLB | FANCC | MAP2K4 | PRDM1 | TNFAIP3 |
| CCND1 | FANCD2 | MAP3K1 | PREX2 | TNFRSF11A |
| CCNE1 | FANCE | MAP3K4 | PRF1 | TNFRSF14 |
| CD274 (PD-L1) | FANCF | MAX | PRKACA | TNFRSF19 |
| CD74 | FANCG | MCL1 | PRKAR1A | TNFSF11 |
| CDA | FANCI | MDM2 | PRKCI | TOP1 |
| CDC73 | FANCL | MDM4 | PRKDC | TOP2A |
| CDH1 | FANCM | MECOM | PRSS1 | TP53 |
| CDK10 | FAT1 | MED12 | PRSS3 | TP63 |
| CDK12 | FBXW7 | MEF2B | PTCH1 | TPMT |
| CDK4 | FGF19 | MEN1 | PTEN | TSC1 |
| CDK6 | FGFR1 | MET | PTK2 | TSC2 |
| CDK8 | FGFR2 | MGMT | PTPN11 | TSHR |
| CDKN1A | FGFR3 | MITF | PTPN13 | TTF1 |
| CDKN1B | FGFR4 | MLH1 | QKI | TUBB3 |
| CDKN1C | FH | MLH3 | RAC1 | TYMS |
| CDKN2A | FLCN | MLLT1 | RAC3 | U2AF1 |
| CDKN2B | FLT1 (VEGFR1) | MLLT3 | RAD50 | UGT1A1 |
| CDKN2C | FLT3 | MLLT4 | RAD51 | VAMP2 |
| CEBPA | FLT4 | MPL | RAD51B | VEGFA |
| CEP57 | FOXA1 | MRE11A | RAD51C | VHL |
| CHD4 | FOXL2 | MSH2 | RAD51D | WAS |
| CHD8 | FOXP1 | MSH6 | RAD54L | WISP3 |
| CHEK1 | FRG1 | MTHFR | RAF1 | WRN |
| CHEK2 | GATA1 | MTOR | RARA | WT1 |
| CREBBP | GATA2 | MUTYH | RARG | XPA |
| CRKL | GATA3 | MYC | RASGEF1A | XPC |
| CSF1R | GATA4 | MYCL | RB1 | XRCC1 |
| CTCF | GATA6 | MYCN | RECQL4 | XRCC2 |
| CTLA4 | GNA11 | MYD88 | RELN | YAP1 |
| CTNNB1 | GNAQ | MYH9 | RET | ZNF217 |
| CUL3 | GNAS | NAT1 | RHOA | ZNF703 |

| **Table S2. Characteristics of *KRAS*-mutated CRC patients** | |
| --- | --- |
| Characteristics | Patients (N=116) |
| Age, median (range), y | 61 (25–79) |
| Age group, No. (%) |  |
| <60 | 53 (45.7) |
| ≥60 | 63 (54.3) |
| Sex, No. (%) |  |
| Female | 57 (49.1) |
| Male | 59 (50.9) |
| Position, No. (%) |  |
| Left-sided colon | 12 (10.3) |
| Right-sided colon | 20 (17.2) |
| Rectum | 40 (34.5) |
| Multiple | 3 (2.6) |
| Unknown | 41 (35.3) |
| Stage at initial diagnosis, No. (%) |  |
| I | 6 (5.2) |
| II | 22 (19.0) |
| III | 40 (34.5) |
| IV | 23 (19.8) |
| Unknown | 25 (21.6) |
| *KRAS* mutation, No. (%) |  |
| G12X | 80 (69.0) |
| G13X | 18 (15.5) |
| A146X | 7 (6.0) |
| Q61X | 5 (4.3) |
| Amplification | 5 (4.3) |
| c.*5−1G>A | 1 (0.9) |
| Microsatellite stability, No. (%) |  |
| Stable | 110 (94.8) |
| Instable | 4 (3.4) |
| Unknown | 2 (1.7) |
| First-line treatment, No. (%) |  |
| Chemotherapy alone | 50 (43.1) |
| Chemotherapy & anti-VEGF | 41 (35.3) |
| Chemotherapy & anti-EGFR | 5 (4.3) |
| Chemoradiotherapy | 5 (4.3) |
| Chemoradiotherapy & anti-VEGF | 2 (1.7) |
| Immunotherapy | 3 (2.6) |
| Unknown | 10 (8.6) |
| Conversion surgery, No. (%) |  |
| With | 59 (50.9) |
| Without | 46 (39.7) |
| Unknown | 11 (9.5) |

CRC: colorectal cancer, VEGF: vascular endothelial growth factor, EGFR: epidermal growth factor receptor

| **Table S3. Characteristics of *KRAS* wild-type CRC patients** | |
| --- | --- |
| Characteristics | Patients (N=73) |
| Age, median (range), y | 59 (24–80) |
| Age group, No. (%) |  |
| <60 | 40(54.8) |
| ≥60 | 33 (45.2) |
| Sex, No. (%) |  |
| Female | 26 (35.6) |
| Male | 47 (64.4) |
| Position, No. (%) |  |
| Left-sided colon | 34 (46.6) |
| Right-sided colon | 15 (20.5) |
| Rectum | 21 (28.8) |
| Unknown | 3 (4.1) |
| Stage at initial diagnosis, No. (%) |  |
| I | 2 (2.7) |
| II | 8 (11.0) |
| III | 23 (31.5) |
| IV | 37 (50.7) |
| Unknown | 3 (4.1) |
| Microsatellite stability, No. (%) |  |
| Stable | 40 (54.8) |
| Instable | 5 (6.8) |
| Unknown | 28 (38.4) |
| First-line treatment, No. (%) |  |
| Chemotherapy alone | 27 (37.0) |
| Chemotherapy & anti-VEGF | 23 (31.5) |
| Chemoradiotherapy | 1 (1.4) |
| Chemoradiotherapy & anti-VEGF | 4 (5.5) |
| Unknown | 6 (6.2) |
| Without progression after radical surgery | 12 (16.4) |
| Conversion surgery, No. (%) |  |
| With | 14 (19.2) |
| Without | 41 (56.2) |
| Unknown | 6 (8.2) |
| Without progression after radical surgery | 12 (16.4) |

CRC: colorectal cancer, VEGF: vascular endothelial growth factor

**Supplementary Figure captions**

**Figure S1. Colorectal cancer inclusion and genomic profiles of *KRAS*-mutated colorectal cancer.**

(A) A total of 116 *KRAS*-mutated and 73 *KRAS* wild-type colorectal cancer patients with tissue or liquid biopsies were included. (B) The genomic profiles of *KRAS*-mutated colorectal cancer patients. (D) The prevalence of mutated RTK signaling pathway in patients with *KRAS^G12X^*, *KRAS^G13X^,* and *KRAS* aberrations other than *KRAS^G12X^* and *KRAS^G13X^* (the Other subgroup).

**Figure S2. Genomic profiles of *KRAS* wild-type colorectal cancer.**

(A) The genomic profile of *KRAS* wild-type colorectal cancer. (B) (C) Higher prevalence of mutated RAS signaling pathway and *BRAF* gene in *KRAS* wild-type colorectal cancer than in *KRAS*-mutated colorectal cancer.

**Figure S3. Mutational signatures detected in *KRAS*-mutated colorectal cancer.**

Mutational signatures were extracted using the R package *sigminer* (version 2.1.3), and the mutational signature exposure in percentage (%) of each eligible *KRAS*-mutated sample was classified according to COSMIC Mutational Signatures version 2 (https://cancer.sanger.ac.uk/signatures/signatures_v2/). Mutational signatures 6, 15, 20, 21, and 26 were defined as DNA mismatch repair deficiency-related mutational signatures.

**Figure S4. Differences in overall survival across colorectal cancer subgroups, and the association between the DNA mismatch repair deficiency signature combination and overall survival.**

(A) Similar overall survival was observed across three subgroups of *KRAS*-mutated patients. (B) Higher prevalence of the DNA mismatch repair deficiency signature combination in patients with mutated TGFβ pathway than patients without. (C) The DNA mismatch repair deficiency signature combination was strongly associated with inferior progression-free survival when adjusting for sex, patient age, *KRAS* mutation subtype, colorectal cancer side, first line regimen, and conversion surgery. (D) The DNA mismatch repair deficiency signature combination was strongly associated with inferior overall survival when adjusting for sex, patient age, *KRAS* mutation subtype, colorectal cancer side, first line regimen, and conversion surgery.

**Figure S5. Differences in overall survival and progression-free survival across colorectal cancer subgroups.**

(A) *KRAS^G12V^* patients had worse overall survival than *KRAS^G12D^* patients. (B) For *KRAS^G12X^* patients receiving chemotherapy alone in the first line treatment, patients with *KRAS^G12X^* other than *KRAS^G12D^* and *KRAS^G12V^* might have worse progression-free survival than *KRAS^G12D^* patients. (C) For *KRAS^G12X^* patients receiving chemotherapy combined with anti- vascular endothelial growth factor agents in the first line treatment, no significant differences in progression-free survival were observed. (D) Mutational signature 15 appeared to be enriched in *KRAS^G12V^* patients.

**Figure S6.** **The DNA mismatch repair deficiency signature combination was not associated with first line therapy progression-free survival or overall survival among patients with *KRAS* mutations other than *KRAS^G12X^*.**

**Supplementary Material and Methods**

**Patients and study design**

Colorectal cancer (CRC) patients diagnosed at the First Hospital of China Medical University between March 2017 and August 2022 were retrospectively enrolled in this study. The inclusion criteria were as follows: (1) adults aged ≥18 years; (2) initially diagnosed with CRC by histopathological examination and radiography methods; (3) with available tumor tissue/liquid biopsies prior to systemic treatment; (4) received regular follow-up. According to the subtype of *KRAS* alterations, *KRAS*-mutated CRC patients were further classified into subgroups for comparisons and subgroup analyses. The molecular features of *KRAS*-mutated CRC were also compared to *KRAS* wild-type CRC. Demographics and clinical information, including age, sex, clinical stage, treatment history, and survival data were obtained from the Hospital Information System of the First Hospital of China Medical University. This study was approved by the Ethics Committee of the First Hospital of China Medical University (Approval No. [2017]-236). All enrolled patients have provided their written informed consent to participate in this study.

**DNA extraction, library preparation, and next generation sequencing (NGS) data processing**

10 mL peripheral blood was collected and centrifuged (1800x g, 10 minutes, at room temperature) within two hours to separate plasma. Circulating free DNA and tumor tissues genomic DNA were extracted from plasma fraction and formalin-fixed, paraffin-embedded (FFPE) tumor tissue samples, respectively, using Qiagen QIAamp Circulating Nucleic Acid Kit or QIAamp DNA FFPE Tissue Kit (Qiagen, Dusseldorf, Germany). After purification, qualification (Nanodrop2000, Thermo Fisher Scientific, Waltham, MA, USA), and quantification (dsDNA HS Assay Kit, Life Technologies, Waltham, MA, USA), sequencing libraries were prepared using KAPA Hyper Prep kit (KAPA Biosystems, Wilmington, MA, USA) with an optimized manufacturer’s protocol. Customized xGen lockdown probes targeting 425 cancer-relevant genes (GeneseeqPrime™, Nanjing Geneseeq Technology Inc., Nanjing, China) were used for hybridization enrichment. Enriched libraries were sequenced on Illumina sequencing platforms (Illumina, San Diego, CA, USA). Sequencing data were analyzed as previously described ^1^. For liquid biopsies, single nucleotide variants and indels with the variant allele frequency (VAF) ≥0.3% and ≥3 unique mutant reads were retained. For tissue biopsies, single nucleotide variants and indels with VAF ≥0.3% and ≥3 unique mutant reads were retained. Copy number variations (CNV) with a fold change ≥1.6 and ≤0.6 were identified as CNV amplification and CNV deletion, respectively.

**DNA mismatch repair deficiency (dMMR)-related mutational signatures**

Mutational signature based on the base-pair changes can reflect the processes of somatic mutation generation, such as aging, smoking, ultraviolet radiation, defects in DNA repair machinery, etc. ^2-5^. Hypermutability and microsatellite instability can result from the deficiencies of the mismatch repair system, which functions in genomic integrity maintenance, and the detection of dMMR can aid in identifying patients who were likely to have aggressive clinical outcomes and response to immunotherapy ^6^. Mutational signatures related to homologous recombination deficiency and dMMR were more common in patients with brain metastases of CRC than in those with primary CRC ^7^. A previous study including 535 CRC samples from The Cancer Genome Atlas demonstrated the detection of dMMR-related mutational signatures 6, 15, and 20, and patients classified into the dMMR-related signature cluster had worse OS than patients classified into the age and tobacco relevant signature cluster ^8^.

In this study, among samples with ≥5 synonymous and/or nonsynonymous single nucleotide variants ^9^, mutational signatures were extracted using the R package *sigminer* (version 2.1.3) ^10^ based on both synonymous and nonsynonymous single nucleotide variants. Mutational signatures were then categorized according to etiology (COSMIC Mutational Signatures version 2, https://cancer.sanger.ac.uk/signatures/signatures_v2/), and mutational signatures 6, 15, 20, 21, and 26 were defined as dMMR-related mutational signatures. Samples identified with <5 synonymous and/or nonsynonymous single nucleotide variants were excluded from mutational signature identification and further analysis involving mutational signatures ^9^.

**Statistical analysis**

Progression-free survival (PFS) was defined as the period from the treatment initiation to radiological progression. Overall survival (OS) of CRC patients with advanced disease was defined as the time from the diagnosis of advanced disease to death from any cause. Fisher’s exact test and two-sample test were performed to test the differences of frequencies and means, respectively. The median follow-up time was calculated using the reverse Kaplan-Meier method ^11^. For survival analysis, Kaplan-Meier curves were generated with differences compared by log-rank tests, and Cox proportional hazards models were fitted to estimate hazard ratios with 95% confidence intervals. The proportionality of hazards was assessed using log(-log) survival plots. For each analysis, individuals with missing data were excluded. All quoted *P*-values were two-tailed, with *P*-values <0.05 as statistically significant. Data were analyzed using R software (version 4.0.3), and the *epiR*, *prodlim*, and *survival* packages.

**References**

1. Shu Y, Wu X, Tong X, et al. Circulating tumor DNA mutation profiling by targeted next generation sequencing provides guidance for personalized treatments in multiple cancer types. *Scientific reports.* 2017;7(1):1-11.

2. Nik-Zainal S, Alexandrov LB, Wedge DC, et al. Mutational processes molding the genomes of 21 breast cancers. *Cell.* 2012;149(5):979-993.

3. Nik-Zainal S, Kucab JE, Morganella S, et al. The genome as a record of environmental exposure. *Mutagenesis.* 2015;30(6):763-770.

4. Meier B, Volkova NV, Hong Y, et al. Mutational signatures of DNA mismatch repair deficiency in C. elegans and human cancers. *Genome research.* 2018;28(5):666-675.

5. Haradhvala N, Kim J, Maruvka Y, et al. Distinct mutational signatures characterize concurrent loss of polymerase proofreading and mismatch repair. *Nature communications.* 2018;9(1):1746.

6. Olave MC, Graham RP. Mismatch repair deficiency: The what, how and why it is important. *Genes, Chromosomes and Cancer.* 2022;61(6):314-321.

7. Sun J, Wang C, Zhang Y, et al. Genomic signatures reveal DNA damage response deficiency in colorectal cancer brain metastases. *Nature communications.* 2019;10(1):3190.

8. Liu Z, Zhang Y, Dang Q, et al. Genomic alteration characterization in colorectal cancer identifies a prognostic and metastasis biomarker: FAM83A| IDO1. *Frontiers in Oncology.* 2021;11:632430.

9. Selenica P, Marra A, Choudhury N, et al. APOBEC mutagenesis, kataegis, chromothripsis in EGFR-mutant osimertinib-resistant lung adenocarcinomas. *Annals of oncology.* 2022;33(12):1284-1295.

10. Wang S, Tao Z, Wu T, Liu X-S. Sigflow: an automated and comprehensive pipeline for cancer genome mutational signature analysis. *Bioinformatics.* 2021;37(11):1590-1592.

11. Schemper M, Smith TL. A note on quantifying follow‐up in studies of. *Control clin trials.* 1996;17:343-346.
