# Supplementary material for: Genetic feature diversity of KRAS-mutated colorectal cancer and the negative association of DNA mismatch repair deficiency relevant mutational signatures with prognosis
Source: Genes Dis. 2024 Feb 26;12(1):101245. doi: 10.1016/j.gendis.2024.101245 (PMC12053584; doi:10.1016/j.gendis.2024.101245)
Supplement: Multimedia component 2 [file mmc2.pdf]

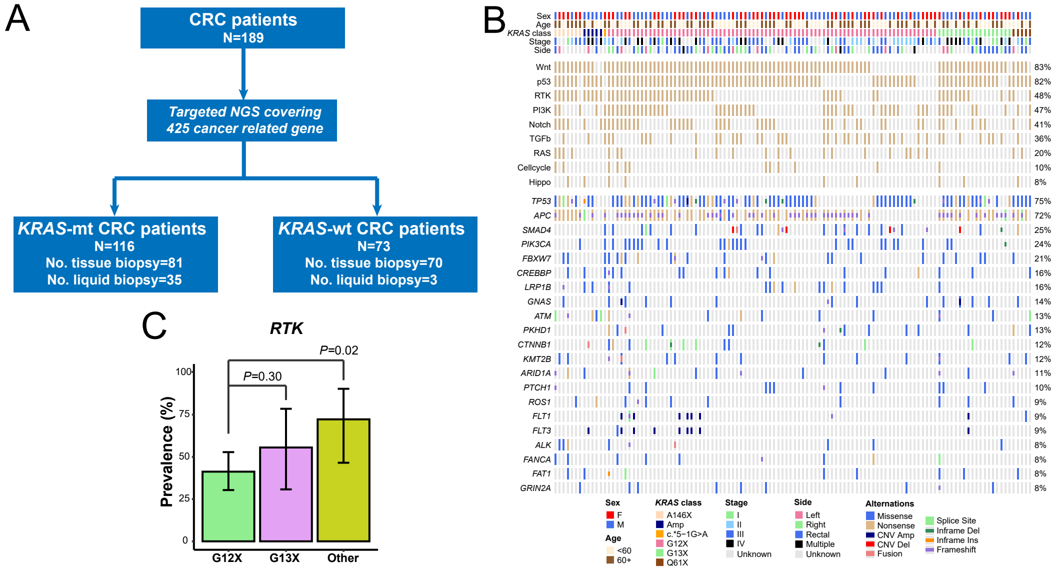
A
CRC patients
N=189
Targeted NGS covering
425 cancer related gene
KRAS-mt CRC patients
N=116
No. tissue biopsy=81
No. liquid biopsy=35
KRAS-wt CRC patients
N=73
No. tissue biopsy=70
No. liquid biopsy=3
B
Sex
Age
KRAS class
Stage
Side
Wnt 83%
p53 82%
RTK 48%
PI3K 47%
Notch 41%
TGFb 36%
RAS 20%
Cellcycle 10%
Hippo 8%
TP53 75%
APC 72%
SMAD4 25%
PIK3CA 24%
FBXW7 21%
CREBBP 16%
LRP1B 16%
GNAS 14%
ATM 13%
PKHD1 13%
CTNNB1 12%
KMT2B 12%
ARID1A 11%
PTCH1 10%
ROS1 9%
FLT1 9%
FLT3 9%
ALK 8%
FANCA 8%
FAT1 8%
GRIN2A 8%
Sex: F, M
Age: <60, 60+
KRAS class: A146X, Amp, c.*5−1G>A, G12X, G13X, Q61X
Stage: I, II, III, IV, Unknown
Side: Left, Right, Rectal, Multiple, Unknown
Alternations: Missense, Nonsense, CNV Amp, CNV Del, Fusion, Splice Site, Inframe Del, Inframe Ins, Frameshift
C
RTK
P=0.02
P=0.30
Prevalence (%)
100
75
50
25
0
G12X
G13X
Other
