# Supplementary figures and images for: Genetic feature diversity of KRAS-mutated colorectal cancer and the negative association of DNA mismatch repair deficiency relevant mutational signatures with prognosis
Source: Genes Dis. 2024 Feb 26;12(1):101245. doi: 10.1016/j.gendis.2024.101245 (PMC12053584; doi:10.1016/j.gendis.2024.101245)

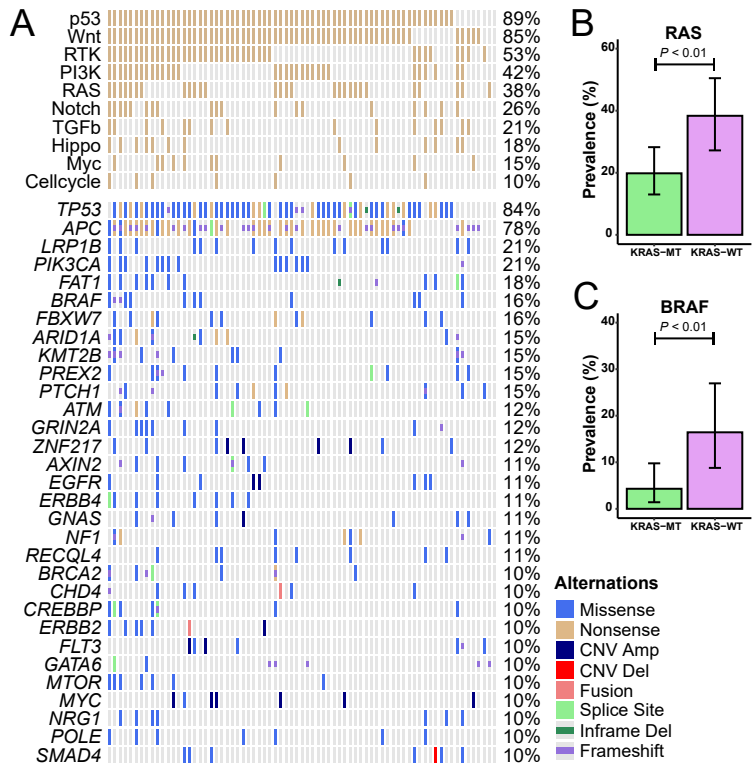

Supplement: Multimedia component 3 [file mmc3.pdf]

# Signatures

dMMR

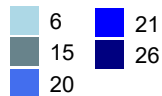

Aging

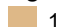

HRD defect

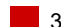

AID/APOBEC

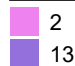

Tobacco

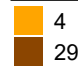

POLE

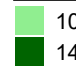

Other

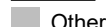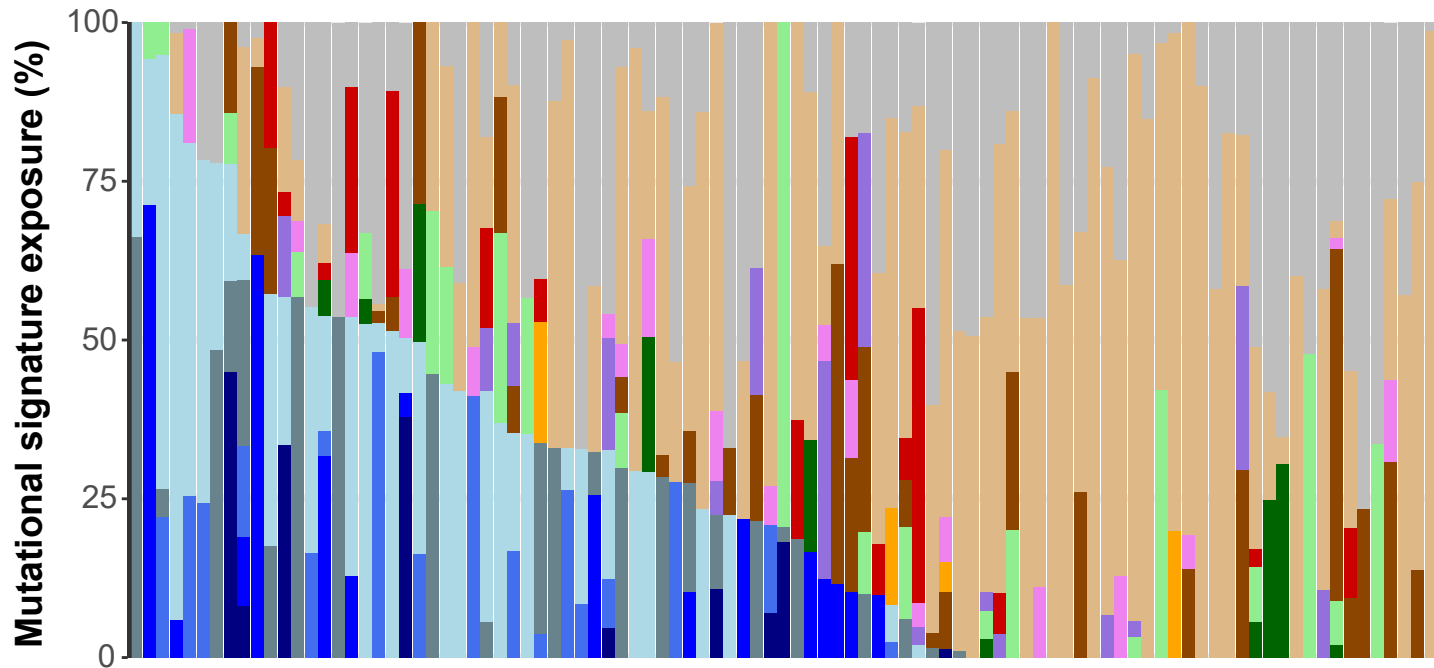

Supplement: Multimedia component 4 [file mmc4.pdf]

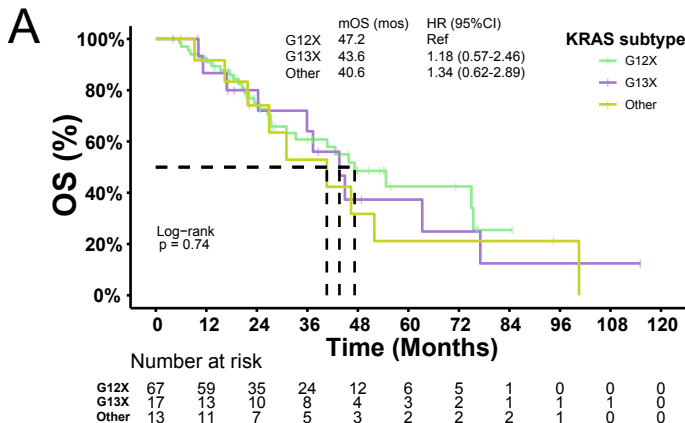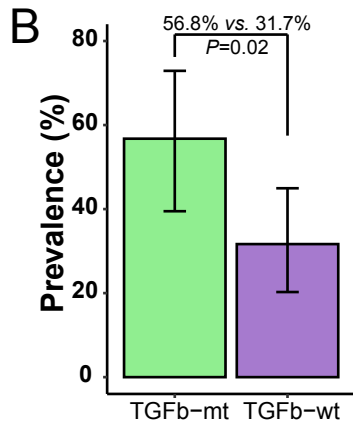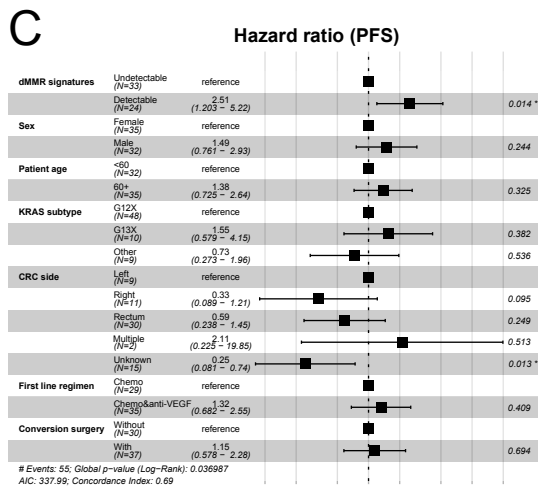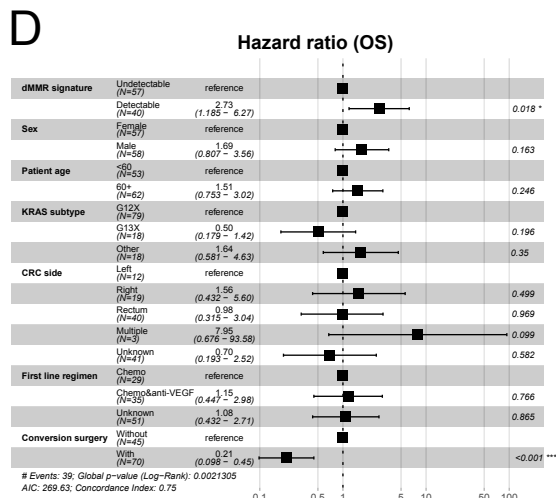

Supplement: Multimedia component 5 [file mmc5.pdf]

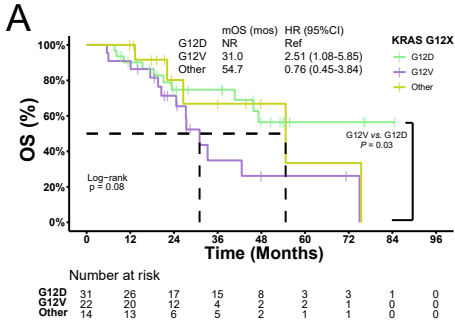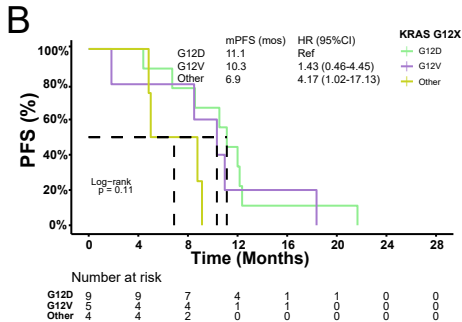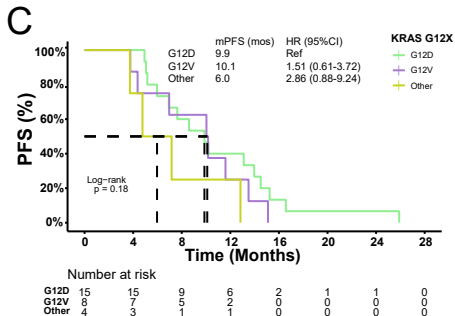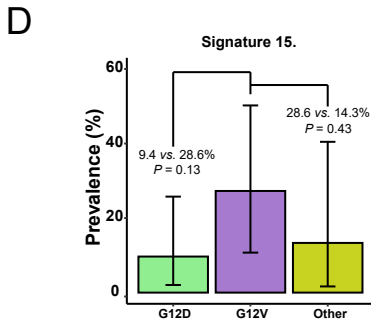

Supplement: Multimedia component 6 [file mmc6.pdf]

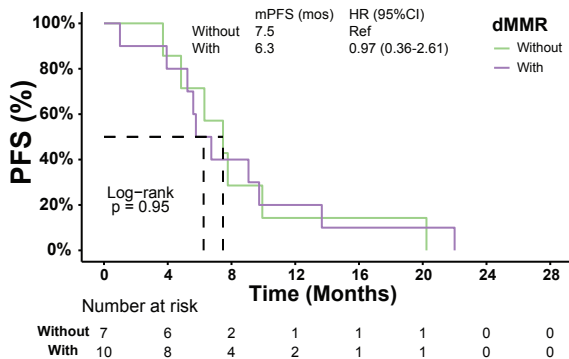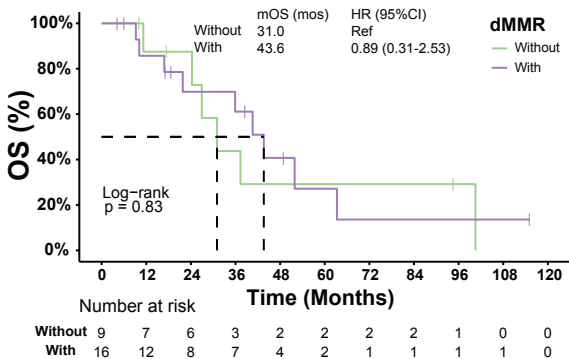

Supplement: Multimedia component 7 [file mmc7.pdf]
